# The Community Structure of the Global Corporate Network

**DOI:** 10.1371/journal.pone.0104655

**Published:** 2014-08-15

**Authors:** Stefania Vitali, Stefano Battiston

**Affiliations:** 1 Department of Banking and Finance, University of Zurich, Zurich, Switzerland; 2 Dipartimento di Scienze Economiche e Sociali, Università Politecnica delle Marche, Ancona, Italy; University of Namur, Belgium

## Abstract

We investigate the community structure of the global ownership network of transnational corporations. We find a pronounced organization in communities that cannot be explained by randomness. Despite the global character of this network, communities reflect first of all the geographical location of firms, while the industrial sector plays only a marginal role. We also analyze the meta-network in which the nodes are the communities and the links are obtained by aggregating the links among firms belonging to pairs of communities. We analyze the network centrality of the top 50 communities and we provide a quantitative assessment of the financial sector role in connecting the global economy.

## Introduction

A recent work has studied the global structure of ownership network with respect to the issue of the corporate control [1]. In this paper, instead, we carry out an in-depth community analysis [2] of the same network, in order to address questions concerning the level of geographical integration and the role of the financial sector in the global economy. To our knowledge this is the first investigation of communities in large-scale economic networks.

An economic network is a structure in which some economic actors, represented as nodes, are connected to some other actors by means of relationships of several types. Previous empirical studies in socio-economic networks, that are relevant for our work, include those focusing on: international trade [3], [4], international financial exposures [5], [6] and financial networks [7]–[13]. More in detail, previous works on networks relevant to corporate governance include: (i) those on corporate boards, e.g., interlocking directorates [14]–[16], and those on firm ownership [1], [17]–[20]. In general, little attention has been devoted to the community structure of economic networks, with the remarkable exception of [21], [22]. Apart from the study of [23] on the Italian corporate board and ownership networks, the other community analyses have focused so far on correlation networks in stock markets [24] and in foreign exchange markets [25].

Our study of the transnational corporation (TNC) ownership network reveals that the majority of the corporations take part in the largest connected component but at the same time display a pronounced organization in communities. Only few algorithms in the literature are suitable to the investigation of large networks without imposing constraints on the number of communities [26]. We first perform the community analysis by applying the method of [27] (hereafter, Louvain method) and we further apply the method of the hierarchical map equation [28] (hereafter, Infomap method). In order to asses the robustness of the resulting community partitions, we compare the community structure in the empirical network with the one obtained from a random link formation process, accounting for the constraints on the degree distribution and on the ownership structure [18], [29], [30]. The comparison reveals that for the rewired networks the community structure is quite homogeneous across realizations and differs considerably from the one of the empirical network. This means that the community structure cannot be considered the result of a random pattern of link formation. Furthermore, we find that firms in the same community tend to share similar geographical location and industrial sector classification. However, the country dominance tends to be more pronounced than the sector dominance. These results are replicated when a different community detection method is applied. Indeed, the communities identified by the [28] algorithm, Infomap hereafter, which is based on a flow and information theoretic clustering method, maintain the geographical and sector properties.

Finally, we consider communities as themselves forming a meta-network [31], in which the link between any two given communities reflects the number of ownership relations among firms from the two communities. We assess the importance of each community in the network by using DebtRank, a centrality measure recently introduced in the complex networks literature in the context of economics [9]. In particular, we apply this method to verify whether the financial sector is a major source of connection among different communities. We find that the community centrality and, thus the potential impact that each community has on the others, changes drastically when we exclude from the sample the firms belonging to the financial sector. Such a difference in centrality quantifies the role played by the financial sector in linking communities of firms characterized by different geographic location and industrial sector.

## Materials and Methods

### Data

The dataset we investigate in this paper is the same that was analyzed in [1] and extracted, by means of the procedure explained in the following, from the Orbis 2007 database (URL: http://www.bvdep.com/en/ORBIS) containing information as of the last quarter of 2007 for more than 30 million economic actors (firms and shareholders). It includes the name of firms, their geographical localization (country and city), industrial classification (NACE) and several financial data. Moreover, the database includes data on about 12 million ownership relations, with information on the name of the shareholder and the amount of shares.

The procedure of extraction is an important part of the methods and it works as follows. We first identify all the transnational corporations (TNC), defined as those companies that are headquartered in one country and operate in at least one foreign country, by owning partially, at least 10% of the shares, or wholly other companies [32]. We obtain a list of 43060 TNCs, located in 115 different countries. The major part of these TNCs have their headquarters in Europe and the US. Nevertheless, some of them are also located in off-shore countries like Bermuda (with 139 companies) and Cayman Islands (with 40 companies). Then, we explore recursively the neighborhood of the TNC companies in the whole database. Two recursive searches are applied: (i) we proceed downstream by identifying all the participated companies directly and indirectly owned by the TNCs with a breadth-first search procedure; (ii) we proceed upstream with the same procedure in order to find the shareholders that have direct and indirect paths leading to the TNCs. In this way, we assemble a network in which each node is connected to at least one TNC. The resulting network consists of 600508 nodes corresponding to economic entities and 1006987 links corresponding to corporate ownership relations.

In an ownership network, the nodes correspond to economic entities (e.g., companies or people owning equity shares) and the links to ownership relationships connecting them. We recall that a network 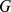 is defined as the set of nodes 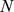 and the set 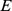 of edges represented by ordered pairs of nodes 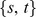, with 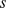 being the source and 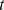 the target nodes of the edge 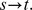 The weighted adjacency matrix of the network is 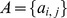, where 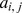 is the share that 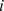 owns in 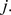 The network does not contain self-loops and the sum of shares of a firm held by other entities can not exceed 100%, i.e., 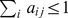 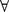 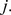 The in-degree, 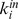, is the number of incoming links to a node 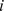, that is, according to the convention we follow here, the number of shareholders. The out-degree, 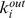, refers, instead, to the number of node 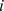
's outgoing links and represents the number of firms in 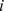
's portfolio. A connected component is a subgraph in which all the nodes can reach all the other nodes via an undirected path.

### Community Detection Procedure

In the field of complex networks, the notion of “community” corresponds, loosely speaking, to a subset of nodes that are more densely connected among themselves than with the nodes outside the subset. Several definitions of community and methods to detect communities have been proposed in the literature (see [2] for a review). Most algorithms can be distinguished in divisive [33], agglomerative [34] and optimization-based [35]. In the latter case, the goodness of the partitions is commonly assessed in terms of the so-called “modularity” [33].

The modularity takes values between 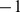 and 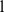 and compares the density of the links within the communities with those across communities. Positive values of modularity are a necessary but not sufficient condition for the presence of communities, since even random graphs can have positive values. Therefore, the values obtained have to be compared with those obtained in ensembles of rewired networks (see more below). Despite the resolution limits of modularity optimization methods [36], especially in detecting small clusters, it has been found to be one of the best methods for network partition. Among the available detection algorithms of the optimization-based class, in this study, we apply the Louvain method which is one of the few methods that are suitable: (i) to analyze large networks with good scalability properties [37] and (ii) to avoid *ex-ante* assumptions on their size [26], [38].

For the sake of robustness, we also run the community analysis with the Infomap algorithm proposed by [28]. Indeed, among the many available ones, this methods has been proved to be one of the best in terms of performance as it is able to process efficiently large networks even in the case of weighted and directed links [37], [38]. This algorithm uses a flow-based and information theoretic clustering method, called the map equation, to uncover the important multilevel structures and their relationships. It determines how many hierarchical levels there are in the network and how many modules are present at each level [28].

Notice that, for the community detection analysis, we have utilized the unweighted and undirected version of the two algorithms described above, for the following reasons. The weight of a link represents a share and, thus, a number between 0 and 1. The weight of links pointing to big firms are typically small, since no single shareholder is able to own (or interested in owning) a large fraction of the capital. Therefore, assigning an importance to the links proportional to the share would result in treating shareholding relations to large firms as very weak links, thus inducing a bias in the communities towards small firms. A more appropriate way to proceed could be to try and account for the monetary value of the links, which depends on the value of the firm owned. Unfortunately, this value is not available in the database for all the firms. Moreover, it would not be clear how to normalize the values in order to use them within the algorithm.

As for the direction of the links, this is clearly crucial in the computation of the potential control of a company on the ones in which it owns shares [1]. However, if we are interested in the structure of groups of economic interest the flow of resources and information is reciprocal and not unidirectional. On the one side, if company A owns shares of company B, then it can influence, with its voting rights and representatives, the decision making process in B's board of directors. The presence of representatives of A in B determines also a flow of information from A to B, so that decisions may spread from a board to another [39]. On the other side, the economic performance of B, especially if it is a subsidiary of A, affects A's profits via its shares. Further, A may shift resources from a subsidiary to another for strategic reasons, depending on the economic performance and the business strategy of A. Therefore, there is also a flow of information from A to B. The community detection method Infomap uses the direction of the links to detect the different levels of hierarchy in the network and, within each level, to further split modules apart. While this may be an interesting question, it is not the focus of our paper. Here, we are interested in communities meant as groups of interest [40]. Therefore, in line with previous community analyses in ownership networks [23], when running the community detection algorithms we consider the network as undirected.

Further, we want to compare the community structure in the empirical network with the one resulting from a random link formation process. In order to account for constraints that arise from the degree distribution, it is custom to generate ensembles of synthetic networks by applying a degree-preserving rewiring procedure [18], [29], [30]. When rewiring the links in an ownership network, it is important to satisfy two main constraints: (i) the degree sequence, i.e., the number of outgoing and ingoing links of each economic entity; and (ii) the total number of ownership shares owned by the shareholders. It is also important to preserve the link directionality in order to not exceed the number of total shares of a company owned by others. Notice that even if we do not use the link weight in the community detection algorithms, it is still important to impose condition (ii) because it correctly constraints the space of possible networks that are generated by the rewiring process.

We, thus, follow the procedure described in [18] (leaving out the additional constraint on the geographical location of firms and shareholders) to generate 20 realizations of synthetic rewired networks.

## Results and Discussion

### Community Structure

**Figure 1:**
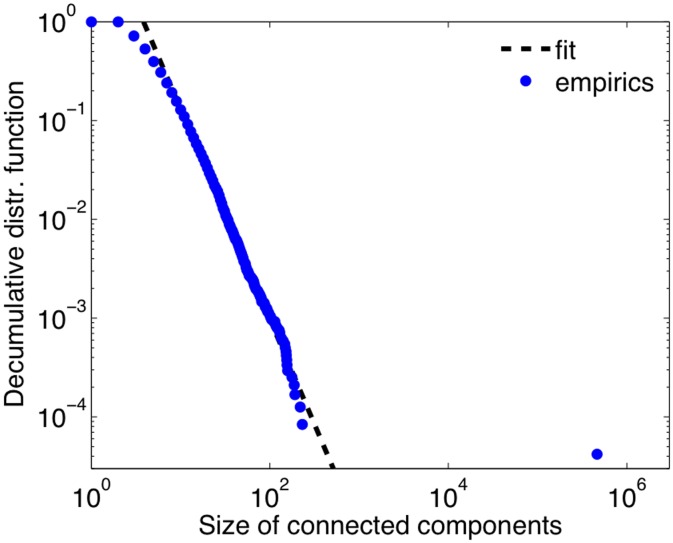
Decumulative distribution function of the size of the connected components. As a comparison, a power-law fit with exponent 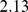 is shown.

The TNC ownership network is composed of 23825 connected components. The largest component contains 463006 nodes (77% of the total), while the second largest connected component contains 230 nodes and 90% of the components have less than 10 nodes. We report in Fig. 1 the distribution of the component size. Notice how the data point corresponding to the largest component deviates from the trend of all the other components. A power law fit of the data points excluding the largest component is shown for reference, yielding an exponent of 2.13.

In the following we have restricted our community analysis to the largest connected component (LCC).

**Figure 2:**
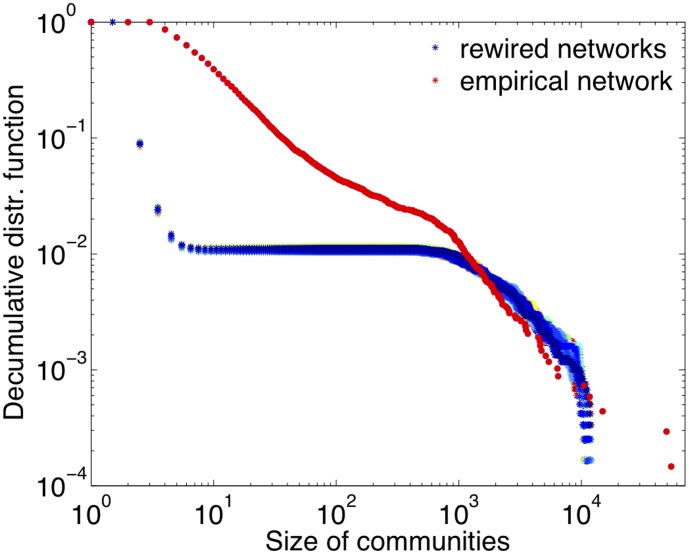
Decumulative distribution function of the size of the communities in the empirical (in red) and in all reshuffled (in blue scale) networks.

In our empirical network, the Louvain algorithm find 6824 communities connected by 25588 links, while the modularity reaches the value of 0.7344. Communities are very heterogeneous in size, ranging from those with only few nodes to the two main communities with about 50 thousands nodes. The 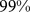 of the communities contains less than 1000 nodes and 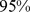 less than 100. The distribution is reported in Fig. 2, in red.

For the rewired networks we find that they are quite homogeneous across realizations, in terms of their community structure, but they differ from the empirical network. They contain, on average, 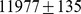 communities, almost the double than the empirical network (6824). The number of links among communities, however, is similar in the synthetic case (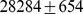) and in the empirical case (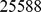). In the empirical network, the value of modularity (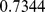) is about 30% larger than in the rewired networks (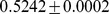). The difference is much larger than 3 times the std in the ensemble of rewired networks. This means that, in terms of modularity, it is very unlikely that the empirical network would occur through a random rewiring process of a network with the same degree sequence and in-weigths. Similarly, the community size distribution in the empirical network (in red) strongly deviates from those obtained from all the rewired networks (in blue color scale, in Fig. 2). We thus conclude that the community structure deviate significantly from the one that would occur by chance in a network with the same characteristics of degree sequence and weights.

### Characterization of Communities in terms of Geography and Sector

In this section we aim to investigate the existence of geographical and sectoral patterns in the detected communities. We start this exercise by analyzing the eight top largest communities, which together comprise 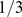 of all the nodes. The results are summarized in Table 1. For each community, the table reports: the number of firms (# firms); (2) the name of the first and the second countries (C1 and C2), and the name of the first and the second sector (Sector1 and Sector2) by number of firms within that community. Moreover, for each community, we sort countries and sectors by their number of firms in the community and we report the percentage of firms for the dominant country and sector – i.e. the top ones by number of firms– (% C1 and % S1). We also report the country and sector concentration, in terms of number of firms, of each community, as measured by the Herfindhal index (Herf. C and Herf. S).

**Table 1:** Statistics on the top 8 communities.

Comm	# firms	Herf. C	%C1	C1	C2	Herf. S	%S1	S1	S2
1	54065	0.362	58.8	US	CA	0.213	25.0	services	manufact.
2	49475	0.208	42.8	GB	DE	0.254	39.5	business act.	services
3	14917	0.578	75.6	ES	GB	0.256	34.5	business act.	services
4	11658	0.669	81.6	FR	GB	0.275	40.6	business act.	services
5	10475	0.685	82.5	DE	GB	0.462	65.3	business act.	financial int.
6	6462	0.539	73.0	IT	DE	0.252	35.2	services	business act.
7	6375	0.411	63.2	DE	GB	0.312	44.6	services	business act.
8	5420	0.265	42.1	BE	NL	0.278	37.8	business act.	financial int.

Herf. C is the Herfindhal index of the country concentration; % C1 is the share of companies localized in the country which dominate the community; C1 is the first dominant country; C2 is the second dominant country; Herf. S is the Herfindhal index of the sector concentration; % S1 is the share of companies active in the sector which dominate the community; S1 is the first dominant sector; S2 is the second dominant sector.

As we can see from the table, the share of firms located in the dominant country is rarely below 0.5 and the Herfindahl index is constantly above the limit between medium and high concentration (i.e., 0.25). However, for some communities the Herfindhal index also reveals that there is more than one dominating country. Indeed, for example in community 2, C1 and C2 contribute roughly equally to the community dominance. The role of geography is evident also in the first 100 biggest communities, which are almost all dominated by a single country located within the North America and Europe boundaries. The first Asian-dominated community is at rank 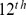 by size (Asian firms, indeed, are dominant only in few communities. The existence of only few and small Asian communities could be due to the traditional organization of Asian corporations in business groups [41], where members are densely connected in relatively small groups with few or no connections with external firms.). For the characterization of communities in terms of sectors, we group all sectors in six macro-sectors (primary, manufacturing, services, financial intermediaries, real estates, renting and business activities, and state and social sectors). The share of the dominant sector in the top eight largest communities is generally smaller than the share of the dominant country, even if the number of possible macro-sectors, i.e. 6, is much smaller than the number of possible countries (Table 1). When we average across all communities with minimum size of 5 nodes, the share of firms belonging to the leading country of each community is 80% in the empirical network as opposed to 25% in the rewired networks. Similarly, the share of firms belonging to the leading sector is 70% against the 35% in the rewired networks.

**Figure 3:**
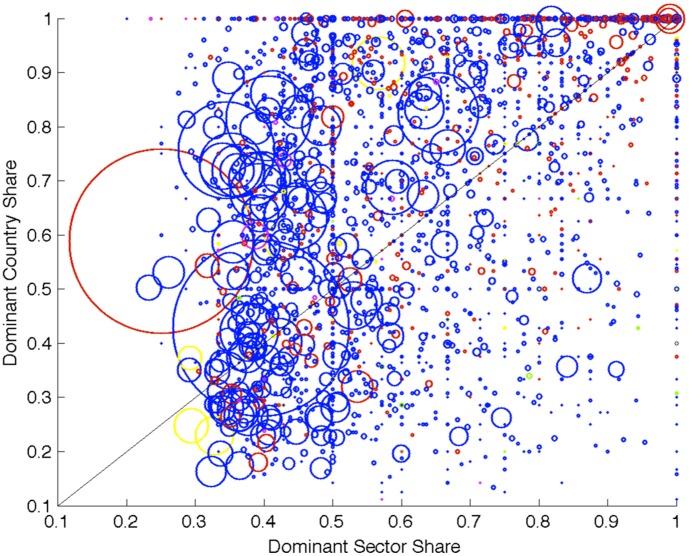
The x and y coordinate of a given circle represent, respectively, the share of firms in the dominant sector and country of a given community. Moving along the x axes corresponds, for a community, to have more firms from the dominant sector. While moving along the y axes corresponds, for a community, to have more firms from the dominant country. For instance, a circle in the top right area represents a community with a large fraction of firms from one sector and one country. The size of the circles is proportional to the number of firms belonging to the community, while the color to the firm localization country (blue for EU, red for North American, yellow for Asian, green for fiscal paradise and magenta for all the other countries). The fact that most circles are located above the diagonal implies that the country dominance tends to be more pronounced than the sector dominance. This is particularly true for small communities.

Fig. 3 illustrates the geography and sector dominance of all the communities. The x and y axes represent the share of firms in the dominant sector and country of the community. The size of the circle reflects the size of the community in log scale and the color reflects the geographical region of the dominant country. As we can see, the fact that most circles are located above the diagonal implies that the country dominance tends to be more pronounced than the sector dominance. Many small communities are completely dominated by a country (value 1 or close to 1 on the y-axis).

Notice also that the two largest communities account together for about 1/5 of all the nodes and comprise companies mainly located in the US and Great Britain, respectively. Here below we provide some more details:

- The first biggest community includes 54065 economic entities. It is dominated by companies mainly located in North America (65%), in particular in the US (59%) and Canada (7%), while 10% of all the firms are located in three Asian countries (Japan, Taiwan and Korea). From a sector point of view, the nodes do not show a unique pattern: roughly 1/4 of the nodes belong, respectively, to the services, manufacturing and real estates, renting and business activities sectors. Finally, even if this community includes only 2283 TNCs (5% of the total), in terms of operating revenue, it represents roughly 34% of the total TNC value.
- The second largest community has 49475 members, of which 2004 TNCs accounting for the 17% of the total operating revenue. Geographically speaking, the nodes belong, almost completely, to European countries (89%), with Great Britain (42%) leading the other countries (Germany is represented by 9.6% of nodes, France by 6%, Sweden by 5% and Italy by 4%.). The largest part of the companies are in the business activity industry (39%), while the services and manufacturing sectors account for 20% and 18% respectively.

Further, we apply the community characterization algorithm introduced in [42]. This statistical method reveals if a particular attribute of a community is “over-expressed”, i.e., if its frequency in the community is larger than what expected from a random occurrence of the attribute across all the nodes in the network. The value of statistical significance level used is 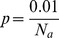, where 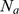 corresponds to the number of observable node attributes, as suggested by the method authors [42]. On the one hand, by taking the location in a country as attribute and considering that in the whole network firms belong to 194 different countries, the algorithm finds that at least one country is over-expressed in all the large communities (i.e. larger than 250 nodes) and in about 50% of the smaller ones. In some communities, especially in the largest ones, more than one country is over-expressed (e.g., in the top two communities), see Table 2. On the other hand, the sector attribute is less over-expressed than the geographical attribute. Indeed, only in roughly 30% of all the communities at least one sector is over-expressed. Only the top ten largest communities display an over-expression of the sector, while many of the smaller community do not. The over-expressed sector for the top 8 largest communities is reported in Table 3). These findings are not replicated when we repeat the analysis for the communities detected in the rewired networks. Indeed, on average, only the 0.28% of the geographical attributes of the communities of these rewired networks is over-estimated, about 65 times less than in the real network communities, where the 20% of the geographical attributes is over-expressed. The same result is worth for the sector attributes, the 0.03% of sectors is over-expressed in the reshuffled network communities, compared to the 15% of the real network ones.

**Table 2:** Statistics of the country characterization across communities.

Comm	# firms	Countries over-expressed
1	54065	US (43.05%) JP (75.08%) CA (41.85%) AU (44.49%) BM (51.81%)
		ZA (40.78%) IN (35.25) KR (25.26%) KY (45.96%) SG (28.67%)
		CN (28.60%) IL (53.42%) BR (21.70%) HK (36.13%) TW (18.47%)
		ID (56.44%) PH (43.11%) TH (29.27%)
2	49475	GB (17.10%) FI (36.12%) SE (20.46%) PL (26.68%) CH (19.88%)
		TH (45.51%) GR (19.37%) NO (14.36%) IT (13.06%) RU (18.01%)
		IE (31.49%) DK (12.98%) LU (14.64%) LI (41.92%) IS (33.33%)
3	14917	ES (52.25%) PT (9.81%) PE (23.75%) AT (5.21%) BR (6.30%)
4	11658	FR (20.50%)
5	10475	DE (14.85%) LU (4.51%)
6	6462	IT (32.28%) RO (57.14%)
7	6375	DE (6.89%) RU (11.83%) CH (6.43%)
8	5420	NL (5.21%) BE (21.27%) LU (4.65%)

The name of each over-expressed country is reported for the top 8 communities. In parentheses, the percentage share of firms belonging to each over-expressed country in the selected community with respect to the total number of firms of that country.

**Table 3:** Statistics of the sector characterization across communities.

Comm	# firms	Sectors over-expressed
1	54065	financial interm. (19.16%) manufacturing (19.91%) primary (26.26%)
		services (12.11%)
2	49475	primary (13.41%) financial interm. (11.45%)
3	14917	services (4.18%) state and social (4.02%) business activities (3.42%)
4	11658	business activities (3.38%) manufacturing (3.15%)
5	10475	business activities (4.71%) financial interm. (3.02%)
6	6462	services (1.79)
7	6375	services (6.35%) state and social (2.09%) primary (2.15%)
8	5420	financial interm. (3.29%) business activities (1.48%)

The name of each over-expressed sector is reported for the top 8 communities. In parenthesis, we report the percentage of firms belonging to each over-expressed sector in the selected community with respect to the total number of firms of that sector.

Overall, the results of these analyses show that the community structure reflects the location of firms in the geographical space, while the role of the sector is much less important.

### Robustness Analysis

In order to investigate the robustness of our findings, we have repeated the analysis by employing the Infomap algorithm, proposed by [28].

The run of this algorithm on the unweighted and undirected ownership network has returned 1180 communities, about 5000 less than the Louvain algorithm, thus larger communities. Indeed, the largest community has about 160000 nodes, about three times the size of the largest one found with the other algorithm (see Table 4). However, we have found that this large community comprises more than the 80% of the nodes of the two largest communities detected with the Louvain algorithm. Such outcome is quite comprehensible because of the hierarchical mapping method of the Infomap algorithm and the pyramid-like structure of the ownership network. Nevertheless, the partitions obtained with the two algorithms are not so dissimilar according to the Mutual Information [43] and the Variation of Information [44] methods. The first one is a measure of the coincidence of two partitions, ranging from 0 -minimum coincidence- to 1 -maximum coincidence-. In our networks, the comparison of the two partitions yields a value of 0.67. The second method is a measure of the lack of information when one aims to infer partition 1 given partition 2, and vice versa. It ranges from 0 -maximum similarity- to 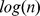 -minimum similarity-, with n being the number of nodes (thus, about 13 in our network), and applied to the Louvain and Infomap partitions it yields a value of 3.29.

**Table 4:** Statistics on the top 8 communities detected with the Infomap algorithm.

Comm	# firms	Herf. C	%C1	C1	C2	Herf. S	%S1	S1	S2
1	162828	0.218	35.9	US	GB	0.222	29.4	business act.	services
2	16042	0.554	30.6	SE	NO	0.259	34.2	business act.	services
3	13152	0.801	74.0	ES	GB	0.262	34.8	business act.	services
4	7925	0.493	89.4	FR	GB	0.276	41.6	business act.	financial int.
5	6169	0.301	69.6	IT	DE	0.239	29.7	business act.	financial int.
6	5845	0.864	52.6	FR	DE	0.304	44.8	business act.	services
7	5521	0.610	92.9	TW	US	0.390	57.0	services	financial int.
8	4536	0.610	76.9	AT	DE	0.415	60.8	business act.	services

Herf. C is the Herfindhal index of the country concentration; % C1 is the share of companies localized in the country which dominate the community; C1 is the first dominant country; C2 is the second dominant country; Herf. S is the Herfindhal index of the sector concentration; % S1 is the share of companies active in the sector which dominate the community; S1 is the first dominant sector; S2 is the second dominant sector.

Furthermore, we have applied the so-called precision-recall method to compare the node partitions found by the Louvain and the Infomap methods. We have assumed the Infomap partition as the benchmark one. In particular, we have computed the F-score (
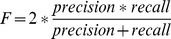
) as follows. Recall is defined as the fraction of nodes in a Louvain community that are also in the same community with the Infomap method. Precision is defined as the fraction of nodes in an Infomap community that also belong to the same community with the Louvain method. F-score values range from 0 (worst score) to 1 (best score). In our analysis, we find 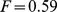, with precision of 0.42 and the recall of 1. This implies that the Infomap algorithm, compared to the “benchmark” Louvain algorithm, returned most of the relevant results, while it also returned both relevant and irrelevant results.

**Figure 4:**
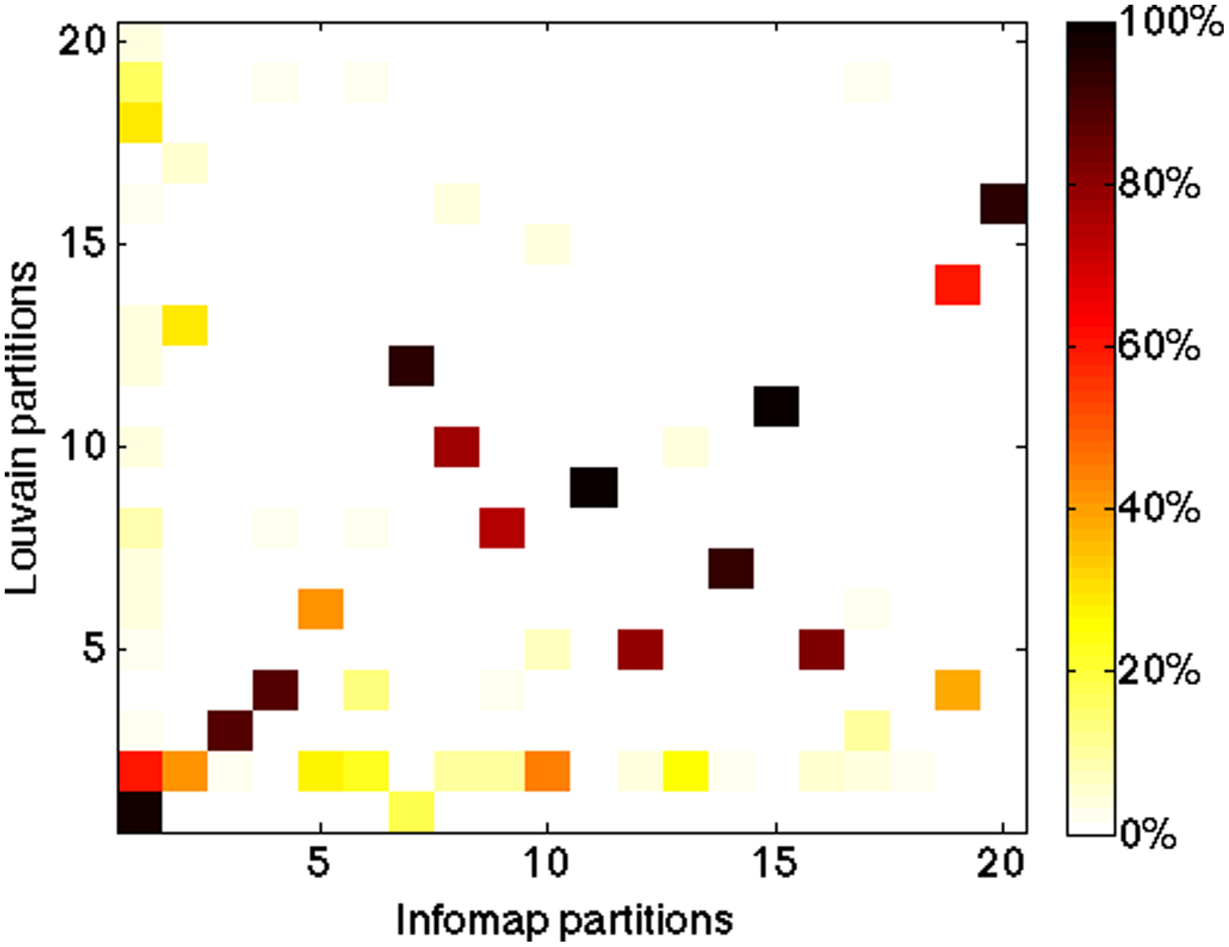
Scatter plot of the top 20 communities with the Louvain (row) and the Infomap (column) algorithms. The color intensity indicates the percentage of nodes that belong, at the same time, to a Louvain and a Infomap community, computed with respect to the minimum size of the two communities.

Finally, we carry out a simple analysis of community similarity by counting the number of firms belonging at the same time to a given Louvain community and to an Infomap community. As shown in Fig. 4, the majority of nodes of the top 20 Louvain communities belong only to one Infomap community.

The community geographical and sector properties are replicated. Indeed, all the communities shows an high geographical concentration, with an Herfindahl index higher than 0.25 in the 82% of the communities, while the share of communities showing an high country dominance (i.e. communities populated by more than 50% by firms belonging to the same country) are about the 80%. The same is true for the sector feature, with the 97% of the communities having a high concentration, according to the Herfindhal index. This patterns are also true for the top 8 communities, as reported in Table 4.

### The Community Meta-Network and the Role of Financial Sector

**Figure 5:**
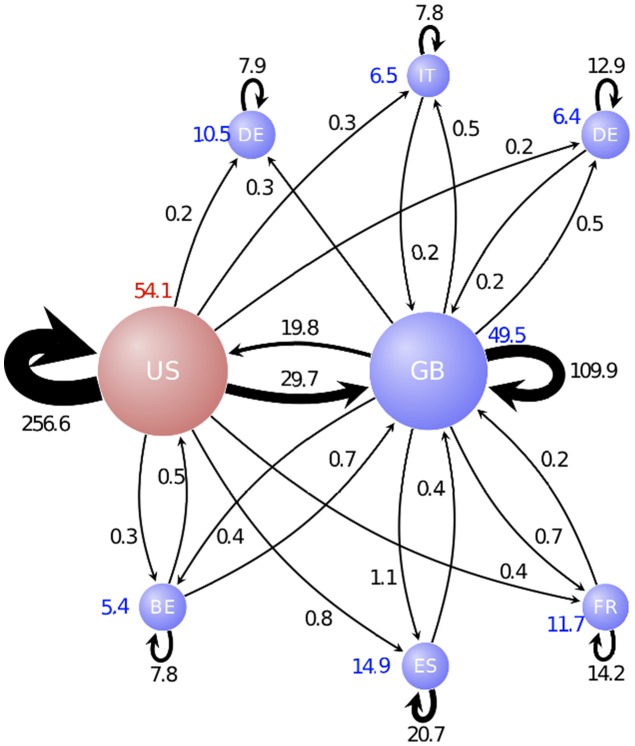
Nodes correspond to the top eight communities and they are labeled with the abbreviation of the dominating countries, colored in blue if EU countries and in red for the US. The node size is proportional to the number of firms populating the community. Edge labels indicate the number of links between communities (in thousand). The links with less than 50 ownership relations have been omitted.

One can also think of communities as the nodes of a network, where the links connecting them are weighted by the number of links between firms in the two communities [29] [31]. The network of the top eight communities by size is shown in Fig. 5.

In order to gain further insight into the whole community network, we start with a basic network analysis. The bow-tie decomposition of the network yields to the largest strongly connected component (LSCC) of 528 nodes, an IN-component of 309 nodes and an OUT-component of 5987. Notice that in the community meta-network the LSCC relative size (7.74%) is much larger than in the firm-shareholder network (1347 nodes out of about 400 thousand, i.e. 0.3% [1]). This has a simple explanation. Consider a community in the OUT. Since links among communities are obtained aggregating links between shareholders and firms, it is enough for that community to have one of its firms investing in a firm belonging to one of the communities in the LSCC to make the community enter the LSCC. However, with a relative size of 7.74%, the LSCC of the meta-network is still much smaller in relative terms than in other paradigmatic real-world networks, such as wikipedia or the world-wide-web [45]. The degree statistics yields: average in-degree 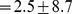, max in-degree 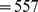, average out-degree 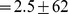, max out-degree 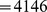. For the shortest paths we find: maximum 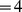; average 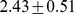. The link density is 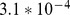.

Financial intermediaries are well integrated in the network and hold many ownership shares in companies belonging to both the non-financial and the financial sector. In fact, in our sample although they represent only a small fraction (9%), at the same time they account for the 36% of all the ownership relations in the network. Many of these relations appear to have a strategic nature. Indeed, the financial intermediaries hold shares larger than 5% in 13% of non-financial companies and in 60% of other financial companies. In order to assess the role of the financial sector as a source of the connections among communities, we repeat the above statistics for the network of communities obtained by removing from each community the firms (46632 in total) that belong to the financial sector (i.e., companies having NACE codes in the classes [6500–7000) and named “financial intermediation, except insurance and pension funding”), and all their links (351587 in total).

The bow-tie decomposition yields now 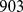 isolated nodes. The LSCC (381 nodes) shrinks by 25%, the IN-component (226 nodes) by 27% and the OUT-component (4799 nodes) by 20%. The degree statistics yields: average in-degree 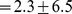, max in-degree 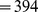, average out-degree 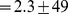, max out-degree 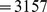. For the shortest paths: maximum 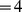; average 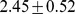. The link density is 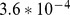. Because some links have been removed, the degree statistics has to decrease and the shortest path statistics has to increase, but the change is small.

As we notice, overall the topology remains close to the case with the financial sector. In contrast, the removal of the financial sector has a strong impact at the level of the weight of the links among communities. Indeed, after removing the firms in financial sector, the number of ownership relations among firms decreases sharply (see Table 5). For instance, in the 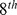 community (the one with the highest share of financial intermediaries), the number of firms decreases by 29% and the number of direct links by 42%. On the other hand, the community less affected by the financial sector is the 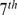, with 3% of removed companies and a reduction of only 340 links. Overall, the links among communities decrease more than proportionally w.r.t. the internal links. Indeed, apart from the 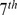 biggest community which counts a small number of financial intermediaries, such result holds for all the communities analyzed. In some cases, the decrease is very strong. For example, the 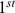 biggest community experiences a drop of 2/3 of the links within itself, and of about 85% of the links directed to the 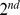 biggest community. This does not happen when we repeat the exercises by considering the other sectors. Indeed, by removing the links between firms, these sectors suffer of a drop in the community connectivity which does not overcome the 30%, with rare exceptions as, for example, the connection between the 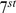 and the 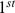 communities, due to the firms in the primary sector dropping by 70% and the connection between the 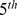 and the 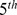 and 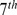 communities, due to the firms in the business activity sector dropping by 80%.

**Table 5:** Statistics on the largest communities with and without financial intermediaries.

	With financial sector			Without financial sector		
*community*	*# firms*	*# rel.*	*density*	*# firms*	*# rel.*	*density*
1	54065	256607	8779e-05	45129	78040	3847e-05
2	49475	109880	4489e-06	44136	52713	2712e-05
3	14917	20799	9348e-05	13529	15726	8892e-05
4	11658	14186	1143e-04	10487	7545	8336e-05
5	10475	12893	1175e-04	9066	6627	1015e-04
6	6462	7812	1711e-04	5781	6541	1530e-04
7	6375	7952	1956e-04	6208	7526	1847e-04
8	5420	7876	2681e-04	3887	3824	1871e-04

The size, the number of directed relations and the density are reported.

Finally, in order to assess the relative importance of each community we use DebtRank, a centrality measure recently introduced in complex networks literature in the context of economic networks [9]. Beyond the interpretation in terms of economic loss due the distress of one or more nodes in the network, DebtRank can be used as a measure of importance, once a network of impact is defined. Here, we define the impact of community 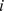 over 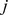 as the ratio between the number of investments of community 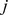 into 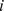 over the number of investments within community 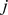, that is 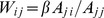, where 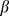 is a rescaling factor that for visualization purposes we set equal to the number of nodes in the network under observation, 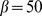. Notice that here we only aim to compare the importance of the communities in the case with and without the financial sector. Traditional measures of centrality are not well suited for this purpose. For instance, Eigenvector Centrality is defined only on strongly connected graphs, or equivalently, on undirected graphs. Other measures of impact, e.g., [10], require a normalization of the impact matrix which then prevents from making an absolute comparison of the importance of a given node across different networks (see [9] for more details).

**Figure 6:**
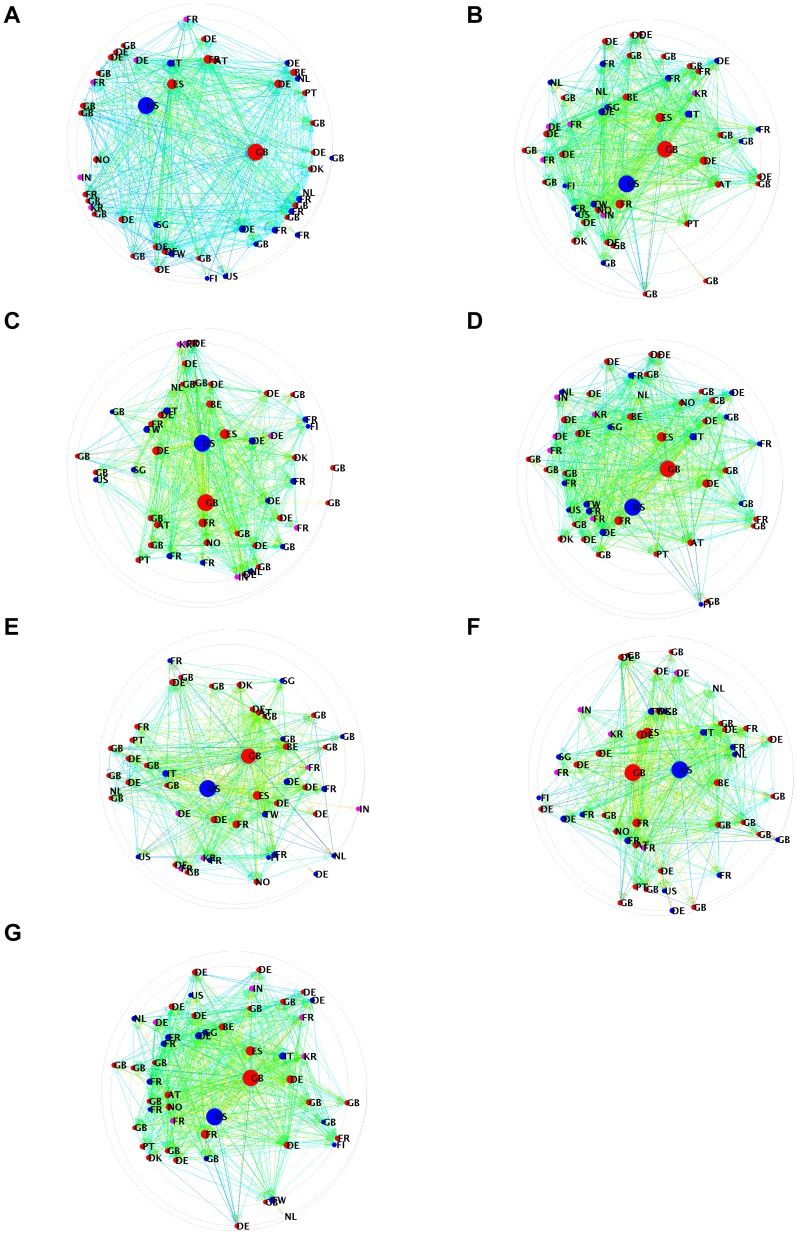
Nodes represent the communities. Outgoing links represent the estimated potential impact of a community to another one. The closer a node is to the center the higher is its DebtRank (e.g., its centrality). The size of the node reflects the number of firms in the community (the size is set larger than a minimum, for visualization purposes). The color of the nodes corresponds to the dominant sector country, as well as the label. The color of a link reflects the DebtRank of the node from which it originates (see [9] for more detail on the figure construction). Community network after removing from the community partition the firms in the financial −46632 firms- (A), in the original dataset (B) and after removing manufacturing −66212 firms- (C), primary −5787 firms- (D), business activities −130587 firms- (E), services −99839 firms- (F) and state and social −21355 firms- (G) sectors.

Fig. 6, B, illustrates the network of the top 50 communities in a diagram where the position of each community reflects its centrality, as measured by DebtRank. More central communities are located in the center of the diagram. The size of each node is proportional to the number of firms in the community, the color corresponds to the dominant sector, while the label indicates the dominant country. As we can expect, the top communities by size are also more central. Fig. 6, A, illustrates the network of the top 50 communities after removing the firms in the financial sector. In this case, the top communities lose much of their centrality. As we can see, while the topological properties that do not account for the weight of the links are only moderately affected by the removal of the financial sector, the centrality computed with DebtRank, which do take weights into account, changes drastically. The difference in centrality quantifies the role played by the financial sector in the strength of the links among communities and, thus, in determining the potential impact that each community has on the others. This change is very marginal when we consider the other sectors. Indeed, as it is possible to observe in Fig. 6, C–G, all the communities remain quite central after removing the firms and links belonging to the sectors: primary, manufacturing, services, real estates, renting and business activities, and state and social.

**Figure 7:**
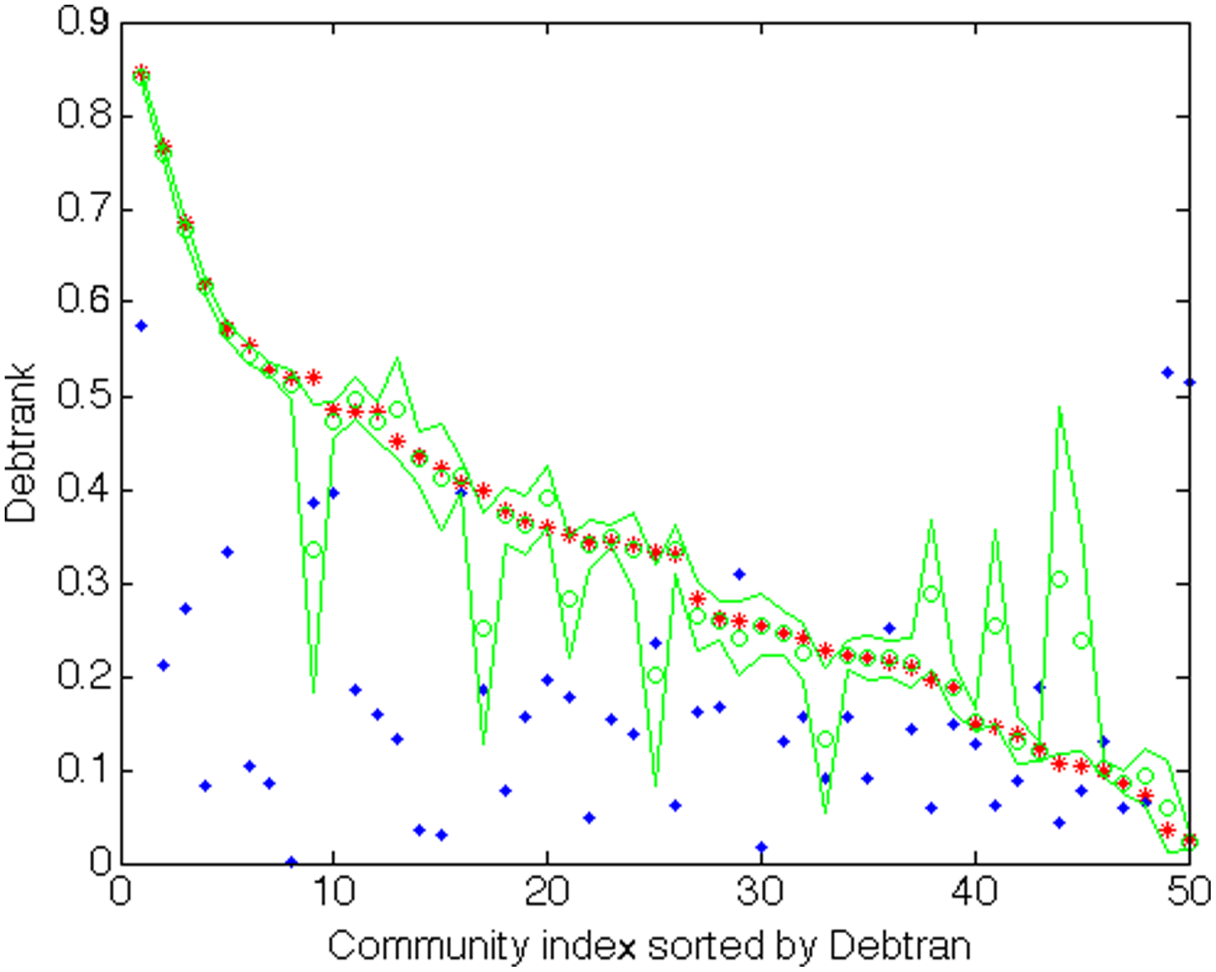
Comparison of Debtrank: the case with no financial sector vs randomized samples. Each curve displays the values of Debtrank (y-axis) across the top communities, sorted in descending order. On the x-axis is the index of the community according to this sorting. The red curve refers to the whole network. The blue dots refer to the network in which the financial sector has been removed. The solid green curve is the average across the 25 randomized samples, while the upper and lower green dashed curves correspond to the average plus or minus one standard deviation of the values on the y-axis in the 25 samples. The figure shows, that removing the financial sector has an effect on the centrality of the community that deviates strongly from the one of randomly removing an equivalent number of firms.

In order to test more directly the effect of removing the financial sector, we recompute the values of DebtRank after randomly removing the same number of firms and links as in the financial sector. We construct 25 such sample and we compute the average (plus and minus the standard deviation) of the DebtRank values of the same communities. Fig. 7 compare the DebtRank values of each of the top 50 communities in the three cases: the whole network (red), the network without the financial sector (blue), the ensemble of networks with randomly removed firms (green). The figure shows, that removing the financial sector has an effect on the centrality of each community that in most cases deviates from the one of randomly removing an equivalent number of firms.

### Concluding Remarks

This paper is a follow up of a previous study on the global corporate network, i.e. the network of ownership among transnational corporations [1]. The present study focuses on the community structure of such network and to our knowledge it is the first investigation of communities in large-scale economic networks. The global corporate network is obtained from a large database of corporate information with a snowball procedure that starts from a list of about 43 thousand transnational corporations and recursively explores all the incoming and outgoing ownership relations building a network of about 600 thousand economic entities.

We have found a pronounced organization in communities that cannot be explained by randomness. Moreover, we find that most communities are characterized by a dominant country, in the sense that the fraction of firms belonging to that country are not only the (relative) majority, but are over-expressed with respect to what would happen if the nationality is distributed at random among the firms. The characterization in terms of sectors is significant, but less pronounced than the one for countries. Thus, we conclude that the global corporate network is strongly clustered in communities, where geography is the major driver while sector is not so important.

We have also analyzed the community meta-network, i.e. the network in which nodes are the communities and links are obtained aggregating the links among the firms belonging to pairs of communities. In order to assess the role of the financial sector in the architecture of the global corporate network, we have analyzed the centrality of the top 50 communities by means of the DebtRank algorithm [9]. This has allowed us to obtain an absolute measure of the importance of each community, which we have then used to compare the case with and without the firms in the financial sector. The difference between these two cases has provided a first quantitative assessment of the role of the financial sector in connecting corporations across countries and sectors.

These findings contribute to the literature about geographic integration and financialization of global economy.
